# WSES/GAIS/WSIS/SIS-E/AAST global clinical pathways for patients with skin and soft tissue infections

**DOI:** 10.1186/s13017-022-00406-2

**Published:** 2022-01-15

**Authors:** Massimo Sartelli, Federico Coccolini, Yoram Kluger, Ervis Agastra, Fikri M. Abu-Zidan, Ashraf El Sayed Abbas, Luca Ansaloni, Abdulrashid Kayode Adesunkanmi, Goran Augustin, Miklosh Bala, Oussama Baraket, Walter L. Biffl, Marco Ceresoli, Elisabetta Cerutti, Osvaldo Chiara, Enrico Cicuttin, Massimo Chiarugi, Raul Coimbra, Daniela Corsi, Francesco Cortese, Yunfeng Cui, Dimitris Damaskos, Nicola de’Angelis, Samir Delibegovic, Zaza Demetrashvili, Belinda De Simone, Stijn W. de Jonge, Stefano Di Bella, Salomone Di Saverio, Therese M. Duane, Paola Fugazzola, Joseph M. Galante, Wagih Ghnnam, George Gkiokas, Carlos Augusto Gomes, Ewen A. Griffiths, Timothy C. Hardcastle, Andreas Hecker, Torsten Herzog, Aleksandar Karamarkovic, Vladimir Khokha, Peter K. Kim, Jae Il Kim, Andrew W. Kirkpatrick, Victor Kong, Renol M. Koshy, Kenji Inaba, Arda Isik, Rao Ivatury, Francesco M. Labricciosa, Yeong Yeh Lee, Ari Leppäniemi, Andrey Litvin, Davide Luppi, Ronald V. Maier, Athanasios Marinis, Sanjay Marwah, Cristian Mesina, Ernest E. Moore, Frederick A. Moore, Ionut Negoi, Iyiade Olaoye, Carlos A. Ordoñez, Mouaqit Ouadii, Andrew B. Peitzman, Gennaro Perrone, Tadeja Pintar, Giuseppe Pipitone, Mauro Podda, Kemal Raşa, Julival Ribeiro, Gabriel Rodrigues, Ines Rubio-Perez, Ibrahima Sall, Norio Sato, Robert G. Sawyer, Vishal G. Shelat, Michael Sugrue, Antonio Tarasconi, Matti Tolonen, Bruno Viaggi, Andrea Celotti, Claudio Casella, Leonardo Pagani, Sameer Dhingra, Gian Luca Baiocchi, Fausto Catena

**Affiliations:** 1 Department of Surgery, Macerata Hospital, Macerata, Italy; 2 Department of General, Emergency and Trauma Surgery, Pisa University Hospital, Pisa, Italy; 3 Department of General Surgery, Rambam Health Care Campus, Haifa, Israel; 4 General Surgery Department, Regional Hospital of Durres, Durres, Albania; 5 Department of Surgery, College of Medicine and Health Sciences, UAE University, Al-Ain, United Arab Emirates; 6 Department of General and Emergency Surgery Faculty of Medicine, Mansoura University Hospital, Mansoura, Egypt; 7 Department of Surgery, Fondazione IRCCS Policlinico San Matteo, University of Pavia, Pavia, Italy; 8 Department of Surgery, Faculty of Clinical Sciences, College of Health Sciences, Obafemi Awolowo University, Ile-Ife, Osun State Nigeria; 9 Department of Surgery, University Hospital Centre Zagreb, Zagreb, Croatia; 10 Trauma and Acute Care Surgery Unit, Hadassah Hebrew University Medical Center, Jerusalem, Israel; 11 Department of General Surgery Bizerte Hospital, Faculty of Medicine of Tunis, University Tunis El Manar, Tunis, Tunisia; 12 Division of Trauma/Acute Care Surgery, Scripps Clinic Medical Group, La Jolla, CA USA; 13 Emergency and General Surgery Department, University of Milan-Bicocca, Milan, Italy; 14 Anesthesia and Transplant Surgical Intensive Care Unit, Ospedali Riuniti, Ancona, Italy; 15 Department of Pathophysiology, ASST Niguarda Ca’Granda Hospital, University of Milano, Milan, Italy; 16 Riverside University Health System, CECORC Research Center, Loma Linda University, Loma Linda, USA; 17 General Direction, Area Vasta 3, ASUR Marche, Macerata, Italy; 18 Emergency Surgery Unit, San Filippo Neri’s Hospital, Rome, Italy; 19 Department of Surgery, Tianjin Nankai Hospital, Nankai Clinical School of Medicine, Tianjin Medical University, Tianjin, China; 20 Department of Surgery, Royal Infirmary of Edinburgh, Edinburgh, UK; 21 Minimally Invasive and Robotic Digestive Surgery Unit, Regional General Hospital F. Miulli, Bari, Italy; 22 Université Paris Est, UPEC, Creteil, France; 23 Department of Surgery, University Clinical Center of Tuzla, Tuzla, Bosnia and Herzegovina; 24 Department General Surgery, Kipshidze Central University Hospital, Tbilisi, Georgia; 25 Department of General, Digestive and Metabolic Minimally Invasive Surgery, Centre Hospitalier Intercommunal De Poissy/St Germain en Laye, Poissy, France; 26 Department of Surgery, Academic Medical Center, University of Amsterdam, Amsterdam, Netherlands; 27 Clinical Department of Medical, Surgical and Health Sciences, Trieste University, Trieste, Italy; 28 Department of General Surgery, “Madonna del Soccorso” San Benedetto del Tronto Hospital, San Benedetto del Tronto, Italy; 29 Department of Surgery, Texas Health Resources, Ft Worth, TX USA; 30 Division of Trauma and Acute Care Surgery, Department of Surgery, University of California Davis, Sacramento, CA USA; 31 Department of General Surgery, Mansoura Faculty of Medicine, Mansoura University, Mansoura, Egypt; 32 Second Department of Surgery, Aretaieion University Hospital, National and Kapodistrian University of Athens, Athens, Greece; 33 Department of Surgery, Hospital Universitário Terezinha de Jesus, Faculdade de Ciências Médicas E da Saúde de Juiz de Fora, Juiz de Fora, Brazil; 34 Department of Upper GI Surgery, Queen Elizabeth Hospital, University Hospitals Birmingham NHS Foundation Trust, Birmingham, UK; 35 Trauma Service, Inkosi Albert Luthuli Central Hospital and Department of Surgery, Nelson R Mandela School of Clinical Medicine, Durban, South Africa; 36 Department of General and Thoracic Surgery, University Hospital Giessen, Giessen, Germany; 37 Department of Surgery, St. Josef Hospital, Ruhr University Bochum, Bochum, Germany; 38 Surgical Clinic “Nikola Spasic”, Faculty of Medicine University of Belgrade, Belgrade, Serbia; 39 Department of Emergency Surgery, City Hospital, Mozyr, Belarus; 40 Department of Surgery, Jacobi Medical Center, Albert Einstein College of Medicine, Bronx, NY USA; 41 Department of Surgery, Ilsan Paik Hospital, Inje University College of Medicine, Goyang, Republic of Korea; 42 General, Acute Care, Abdominal Wall Reconstruction, and Trauma Surgery, Foothills Medical Centre, Calgary, AB Canada; 43 Department of Surgery, Edendale Hospital, Pietermaritzburg, South Africa; 44 Department of General Surgery, University Hospital of Coventry and Warwickshire, Coventry, UK; 45 Division of Trauma and Surgical Critical Care, Department of Surgery, University of Southern California, Los Angeles, CA USA; 46 Department of General Surgery, School of Medicine, Istanbul Medeniyet University, Istanbul, Turkey; 47 Department of Surgery, Virginia Commonwealth University School of Medicine, Richmond, VA USA; 48 Global Alliance for Infections in Surgery, Macerata, Italy; 49 School of Medical Sciences, Universitiy Sains Malaysia, Kota Bharu, Kelantan Malaysia; 50 Abdominal Center, Helsinki University Hospital and University of Helsinki, Helsinki, Finland; 51 Department of Surgical Disciplines, Immanuel Kant Baltic Federal University, Regional Clinical Hospital, Kaliningrad, Russia; 52 Department of General and Emergency Surgery, ASMN, Reggio Emilia, Italy; 53 Department of Surgery, University of Washington, Seattle, WA USA; 54 First Department of Surgery, Tzaneion General Hospital, Piraeus, Greece; 55 Department of Surgery, Post-Graduate Institute of Medical Sciences, Rohtak, India; 56 Second Surgical Clinic, Emergency Hospital of Craiova, Craiova, Romania; 57 Ernest E Moore Shock Trauma Center at Denver Health, Denver, USA; 58 Department of Surgery, Division of Acute Care Surgery, and Center for Sepsis and Critical Illness Research, University of Florida College of Medicine, Gainesville, FL USA; 59 Department of Surgery, Emergency Hospital of Bucharest, Bucharest, Romania; 60 Department of Surgery, University of Ilorin Teaching Hospital, Ilorin, Nigeria; 61 Division of Trauma and Acute Care Surgery, Fundacion Valle del Lili, Cali, Colombia; 62 Department of Surgery, Universidad del Valle, Cali, Colombia; 63 Department of Surgery, Hassan II University Hospital, Medical School of Fez, Sidi Mohamed Benabdellah University, Fez, Morocco; 64 Department of Surgery, University of Pittsburgh School of Medicine, UPMC-Presbyterian, Pittsburgh, USA; 65 Department of Emergency Surgery, Parma Maggiore Hospital, Parma, Italy; 66 Department of Surgery, UMC Ljubljana, Ljubljana, Slovenia; 67 Department of Internal Medicine, Division of Infectious Disease, ARNAS Civico-Di Cristina Hospital, Palermo, Italy; 68 Department of General and Emergency Surgery, Cagliari University Hospital, Cagliari, Italy; 69 Department of Surgery, Anadolu Medical Center, Kocaeli, Turkey; 70 Infection Control, Hospital de Base, Brasília, DF Brazil; 71 Department of General Surgery, Kasturba Medical College and Hospital, Manipal Academy of Higher Education, Manipal, India; 72 General Surgery Department, Colorectal Surgery Unit, La Paz University Hospital, Madrid, Spain; 73 General Surgery Department, Military Teaching Hospital, Dakar, Senegal; 74 Department of Aeromedical Services for Emergency and Trauma Care, Ehime University Graduate School of Medicine, Ehime, Japan; 75 Department of Surgery, Western Michigan University School of Medicine, Kalamazoo, MI USA; 76 Department of General Surgery, Tan Tock Seng Hospital, Singapore, Singapore; 77 Donegal Clinical Research Academy Emergency Surgery Outcome Project, Letterkenny University Hospital, Donegal, Ireland; 78 Department of Anesthesiology, Neuro Intensive Care Unit, Florence Careggi University Hospital, Florence, Italy; 79 Department of Surgery, AAST Cremona, Cremona, Italy; 80 Department of Clinical and Experimental Sciences, University of Brescia, Brescia, Italy; 81 Department of Infectious Diseases, Bolzano Hospital, Bolzano, Italy; 82 Department of Pharmacy Practice, National Institute of Pharmaceutical Education and Research (NIPER), Hajipur, Bihar India; 83 Department of Surgery, “Bufalini” Hospital, Cesena, Italy

**Keywords:** Skin and soft-tissue infections, Necrotizing soft-tissue infections, Necrotizing infections

## Abstract

Skin and soft-tissue infections (SSTIs) encompass a variety of pathological conditions that involve the skin and underlying subcutaneous tissue, fascia, or muscle, ranging from simple superficial infections to severe necrotizing infections.

Together, the World Society of Emergency Surgery, the Global Alliance for Infections in Surgery, the Surgical Infection Society-Europe, The World Surgical Infection Society, and the American Association for the Surgery of Trauma have jointly completed an international multi-society document to promote global standards of care in SSTIs guiding clinicians by describing reasonable approaches to the management of SSTIs.

An extensive non-systematic review was conducted using the PubMed and MEDLINE databases, limited to the English language. The resulting evidence was shared by an international task force with different clinical backgrounds.

## Introduction

Skin and soft-tissue infections (SSTIs) encompass a variety of pathological conditions that involve the skin and underlying subcutaneous tissue, fascia, or muscle, ranging from simple superficial infections to severe necrotizing infections. Necrotizing soft-tissue infections (NSTIs) are rare but potentially life-threatening and disabling infections [1].

About 20–30% of patients with NSTI die during the hospital stay [1]. Moreover, patients with NSTIs may have considerable long-term functional disabilities. In one study, only half of them could return directly home while the others needed further hospitalization or transfer to an inpatient rehabilitation facility [2].

Successful management of patients with NSTIs involves prompt recognition, appropriate antibiotic therapy, timely surgical debridement or drainage, and resuscitation when required. Close cooperation between surgeons, microbiologists, infectious diseases specialists, and intensivists is fundamental in treating severely ill patients with NSTI.

Together, the World Society of Emergency Surgery (WSES), the Global Alliance for Infections in Surgery (GAIS), the Surgical Infection Society-Europe (SIS-E), The World Surgical Infection Society (WSIS), and the American Association for the Surgery of Trauma (AAST) have jointly completed an international multi-society document to promote global standards of care in SSTIs guiding clinicians by describing reasonable approaches to the management of SSTIs.

An extensive non-systematic review was conducted using the PubMed and MEDLINE databases, limited to the English language. The resulting evidence was shared by an international task force with varying backgrounds.

## Classifications

Various classification systems have been used to describe SSTIs, including variables such as anatomic location, causative pathogen(s), rate of progression, depth of infection, and severity of clinical presentation [3–7].

In 1998, the US Food and Drug Administration (FDA) classified SSTIs into two broad categories for clinical trials evaluating new antimicrobials for their treatment: uncomplicated and complicated. Uncomplicated SSTIs included superficial infections such as cellulitis, simple abscesses, impetigo, and furuncles and required antibiotics or surgical incision to drain abscess alone. In contrast, complicated SSTIs included deep soft-tissue infections such as necrotizing infections, infected ulcers, infected burns, and major abscesses, requiring significant surgical intervention with drainage and debridement [3].

In 2003, Eron et al. classified SSTIs [4] according to the severity of local and systemic signs and the presence or absence of comorbid conditions in patients presenting in the outpatient setting to guide the clinical management, treatment, and admission decisions. In this classification system, SSTIs were divided in four classes:

- *Class 1* patients with SSTI, but no signs or symptoms of systemic toxicity or co-morbidities.
- *Class 2* patients are either systemically unwell with stable co-morbidities or systemically well, but with comorbidity (e.g., diabetes, obesity) that may complicate or delay resolution.
- *Class 3* patients appear toxic and unwell (fever, tachycardia, tachypnoea, and/or hypotension).
- *Class 4* patients have sepsis syndrome and life-threatening infection; for example, necrotizing fasciitis.

SSTIs may be classified according to the anatomical tissue layers involved [5]. Superficial infections such as erysipelas, impetigo, folliculitis, furuncles, and carbuncles are located at the epidermal and dermal layers, while cellulitis is located in the dermis and subcutaneous tissue. Deep infections extend below the subcutaneous tissue may involve fascial planes, or muscular compartments presenting as complex abscesses, fasciitis, or myonecrosis. SSTIs may also be classified as non-necrotizing or necrotizing infections.

In 2014, the Infectious Diseases Society of America (IDSA) updated practice guidelines for the diagnosis and management of skin and soft-tissue infections [6]. The guidelines divided infections by purulent and non-purulent, severity (mild, moderate, and severe), and tissue necrosis (necrotizing versus non-necrotizing).

In 2018, the US FDA introduced the new definition of acute bacterial skin and skin structure infection (ABSSSI) to more closely define complicated soft-tissue infection for the purposes of registration trials. ABSSSIs include cellulitis/erysipelas, wound infections, and major cutaneous abscesses. Thus, an ABSSSI is defined by FDA as a bacterial skin infection with a lesion size area of ≥ 75 cm^2^ (lesion size measured by the area of redness, edema, or induration).

In 2015, WSES published its guidelines for the management of SSTIs [7], proposing a new definition dividing SSTIs into three main groups: surgical site infections (SSIs), non-necrotizing SSTIs, and necrotizing SSTIs. SSIs are classified into two subgroups: (a) incisional and (b) organ and organ/space. The incisional SSIs are divided into superficial (skin and subcutaneous tissue) and deep (deep soft-tissue muscle and fascia). Organ and organ/space infections are not truly soft-tissue infections. SSIs represent a separate topic among soft-tissue infections. They are post-operative infections, and they are framed into a separate group because of their multifaceted aspects. Non-necrotizing SSTIs, including erysipelas, impetigo, folliculitis, simple abscess, and a complex abscess, may be treated by antibiotics or drainage alone. Necrotizing SSTIs require surgical intervention, including drainage and debridement of necrotic tissue in addition to antibiotic therapy.

In 2018 an expert panel from the WSES and SIS-E recommended the necrotizing or non-necrotizing character of the infection, the anatomical extension, the characteristics of the infection (purulent or not purulent), and the clinical condition of the patient should always be assessed independently to classify patients with SSTIs [8].

## Principles of treatment

### Principles of antibiotic therapy

The principal barrier against microbial invasion is the skin. It constantly interacts with the external environment and is colonized with different populations of bacteria. Intact and well vascularized skin is highly resistant to bacterial invasion [8].

Most SSTIs involving healthy skin are caused by aerobic Gram-positive cocci, specifically *S. aureus,* and streptococci. Strains of *S. aureus* and group A streptococci (GAS) can produce a variety of toxins that may both potentiate their virulence and affect the soft tissues and allow invasion of the dermis [8]. Polymicrobial infections occur when aerobic Gram-negative and anaerobes invade soft tissues.

A retrospective population-based study [9], analyzed 376,262 individuals experienced 471,550 SSTI episodes, of which 23% were cultured. Among episodes with a culture, 54% had a pathogen identified. *S. aureus* was by far the most common pathogen, isolated in 81% of pathogen-positive index specimens. Among *S. aureus* isolates, 46% were methicillin-resistant *S. aureus* (MRSA). Other important pathogens were beta-hemolytic streptococci and Gram-negative bacteria. SSTIs were associated with older age and diabetes. *S. aureus* infections were associated with younger age, carbuncle and furuncle. MRSA infections were associated with extreme age (older age and age < 5 years), carbuncle and furuncle, cellulitis and abscess.

Considerable variation in the resistance rates of *S. aureus* to methicillin (or oxacillin) in patients with SSTIs has been noted between continents, with the highest rates in North America (35.9%), followed by Latin America (29.4%) and Europe (22.8%) [10]. Although MRSA has been usually acquired during exposure in hospitals and other healthcare facilities, there has been an increase in MRSA infections presenting in the community (CA-MRSA) [8]. CA-MRSA is genetically distinct from hospital-associated MRSA (HA-MRSA) [11], being resistant to fewer non-beta-lactam antibiotics, and often producing a cytotoxin, Panton-Valentine leukocidin (PVL). Methicillin resistance, due to altered penicillin binding protein (PBP-2a), is encoded by the *mecA* gene. Fourteen SCC*mec* sequence types have been reported. Studies showed that CA-MRSA contain SCC*mec* types IV and V, mostly with the PVL gene, while HA-MRSA carry SCC*mec* types I, II and III.

The prevalence of CA-MRSA varies worldwide, ranging from less than 1% in some countries to more than 50% in others, with the prevalence been higher in children than in adults [12–14].

Clindamycin has been used to treat CA-MRSA, although clindamycin resistance is now very common [8]. Like penicillin, clindamycin has activity against group A and B streptococci and *S. aureus*. It has virtually no activity against Gram-negative bacteria. Furthermore, clindamycin is bacteriostatic, and its use is at high risk for the development of *Clostridioides difficile* infection.

Several observational studies and one small randomized trial [15–17] suggest that trimethoprim and sulfamethoxazole (TMP-SMX), doxycycline, and minocycline are effective against CA-MRSA. However, they have no activity against group A and B streptococci. Therefore, if coverage for both streptococci and MRSA is desired for oral therapy, options include clindamycin, linezolid or tedizolid alone or the combination of either TMP-SMX or doxycycline with a beta-lactam agent (amoxicillin, cephalexin) or azithromycin in the case of beta-lactam allergy.

For many years glycopeptides have been the microbiological agents of choice in complicated Gram-positive infections. Frequent use of vancomycin as the drug of choice for treatment of infections caused by MRSA has putatively led to selection of the isolates with reduced susceptibility to vancomycin.

Fortunately, staphylococcal resistance to glycopeptides such as in vancomycin intermediate *S. aureus* (VISA) and in vancomycin resistant *S. aureus* (VRA) remains rare, although rising minimal inhibitory concentrations (MICs) of glycopeptides may affect the efficacy of these antibiotics [18]. Heterogeneous vancomycin intermediate *S. aureus* (hVISA) shows MICs in the susceptible range (≤ 2 μg/mL), because of a sub-population that expresses a resistant phenotype [19]. Infections caused by VRSA, VISA and hVISA can lead to higher rates of vancomycin treatment failure and are associated with extended hospitalization, higher risk of persistent infection, and elevated treatment costs [19].

Increased resistance to glycopeptides alongside with the search for a better tissue penetration and for protein synthesis inhibition has encouraged the development of new agents active against Gram-positive bacteria, particularly for severe soft-tissue infections where aggressive antibiotic management is always recommended, such as linezolid and daptomycin. Linezolid has been considered an agent of choice in complicated SSTIs. It has the advantages to be a lipophilic drug with possibility of early intravenous-to-oral switch with the oral preparation having very high bioavailability, moreover it inhibits proteic synthesis (for example toxin production inhibition) [20].

Daptomycin has proven efficacy in patients with Gram-positive complicated SSTIs, including those caused by MRSA [21]. Daptomycin has been shown to achieve very good concentrations in the skin and soft tissues and to have a rapid bactericidal effect [21].

Telavancin is a semisynthetic lipoglycopeptide vancomycin-derivative approved by the US FDA in 2009 for the treatment of complicated skin and skin structure infections caused by Gram-positive bacteria, including MRSA (*S. aureus, Streptococcus agalactiae, Streptococcus pyogenes, Streptococcus anginosus* group, or *Enterococcus faeca is*) [22]. Given its extensive binding to plasma proteins, long half-life, and a long post-antibiotic effect, it represents an addition to the therapeutic armamentarium in combating infections caused by resistant Gram-positive pathogens, including MRSA.

Ceftaroline is an oxyimino advanced-generation broad-spectrum cephalosporin which has in vitro activity against both methicillin-susceptible *S. aureus* and MRSA. Ceftaroline fosamil has been found to be similar efficacy compared to vancomycin plus aztreonam for the treatment of cSSTI [23]. Ceftaroline fosamil acetate was also well-tolerated and had a safety profile concordant with other antibiotics in the cephalosporin class.

More recently, new drugs have been approved for ABSSSI and have an important activity against MRSA, especially dalbavancin and tedizolid. Tedizolid, a novel oxazolidinone with Gram-positive activity including MRSA, is promising because it can be administered daily in oral or intravenous forms [24], and dalbavancin, a second-generation lipoglycopeptide that covers MRSA, can be administered intravenously as infrequently as once weekly [25].

Antibiotics recommended for MRSA infections are listed below.

Oral options:

- Minocycline 100 mg every 12 h
- Trimethoprim and sulfamethoxazole 160/800 or 320/1600 every 12 h
- Doxycycline 100 mg every 12 h
- Clindamycin 300–450 mg every 8 h (high resistance rate)
- Linezolid 600 mg every 12 h
- Tedizolid 200 mg every 24 h

Intravenous options:

- Clindamycin 600–900 mg every 8 h
- Trimethoprim and sulfamethoxazole 320/1600 every 12 h
- Vancomycin 25–30 mg/kg loading dose then 15–20 mg/kg/dose every 12 h
- Tigecycline 100 mg IV as a single dose, then 50 mg IV every 12 h
- Linezolid 600 mg every 12 h
- Daptomycin 6 mg/kg every 24 h
- Ceftaroline 600 mg every 12 h
- Dalbavancin 1000 mg once followed by 500 mg after 1 week or 1500 mg one dose
- Tedizolid 200 mg every 24 h
- Telavancin 10 mg/kg every 24 h

### Principles of source control

Source control includes drainage of infected fluids, debridement of infected soft tissues, removal of infected devices or foreign bodies. It should also include definite measures to correct any anatomic derangement resulting in ongoing microbial contamination and restoring optimal function [8]. Appropriate source control is of utmost importance in the management of SSTIs.

Source control is the most important determinant of outcome in NSTIs [8]. Delayed or inadequate source control results in preventable morbidity and mortality in patients with NSTIs [26].

A recent systematic review and meta-analysis [27] demonstrated that mortality was significantly lower for patients with surgery within 6 h after presentation compared to when treatment was delayed more than 6 h (OR 0.43; 95% CI 0.26–0.70; 10 studies included). Surgical treatment within 6 h resulted in a 19% mortality rate compared to 32% when surgical treatment was delayed over 6 h.

Skin grafting and extensive rehabilitation are necessary to mitigate disfigurement after invasive debridements, limited joint mobility, and chronic pain. When debridement focuses only on tissue directly involved in necrosis, viable skin and subcutaneous tissue can remain in place despite wide debridement of deeper tissue planes [28, 29].

There is a lack of literature examining outcomes in NSTIs when surgical re-debridements are performed pre-emptively versus on-demand intervals. However, we believe scheduled re-explorations should be done at least every 12–24 h after the initial operation, at times earlier if clinical local or systemic signs of worsening infection become evident, as well as with worsening laboratory parameters. Re-explorations should be repeated until the wound demonstrates little or no debridement is required. A prospective observational study by Okoye et al. [30] showed that delayed re-debridement after initial source control in necrotizing infections results in worse survival and an increased incidence of acute kidney injury.

## Simple abscess

Cutaneous abscesses are collections of pus within the dermis and deeper tissues.

To be considered a simple abscess, induration and erythema should be limited only to a defined area of the abscess and should not extend beyond its the borders of the abscess. Additionally, simple abscesses should not have extension into deeper tissues or multiloculated extension.

Epidermoid cysts, often named “sebaceous cysts,” are usually due to infection of pilosebaceous gland and are present anywhere in the body lined by squamous epithelium.

Furuncles also named “boils” are superficial infections with suppuration of the hair follicle, usually caused by *S. aureus*. They extend through the dermis into the subcutaneous tissue, where form a small abscess. Furuncles can occur anywhere on hairy skin. A group of infected hair follicles with pus is named carbuncle. Carbuncles are larger and deeper than furuncles. Furuncles often rupture and drain spontaneously or following treatment with moist heat. Most large furuncles and all carbuncles should be treated with incision and drainage. Excision of carbuncle with primary split-thickness skin grafting is an alternative treatment modality.

A recurrent abscess at a site of previous infection may be caused by local causes such as a pilonidal cyst, hidradenitis suppurativa, or foreign material. Therefore, it always requires research of a local cause.

*Treatment*

- Incision and drainage
- Antibiotic therapy only in selected patients for 5 days. You may extend therapy up to 7–10 days if lack of symptom resolution at 5 days

Incision, evacuation of pus and debris, and probing of the cavity to break up loculations provide effective treatment of cutaneous abscesses. The resultant wound should be left open and lightly packed with roll gage soaked with antiseptic solution.

Antibiotic therapy should be prescribed for abscesses greater than 5 cm, in an area difficult to drain (e.g., face, hand, and genitalia), if there is lack of response to incision and drainage alone, if there are multiple localizations and in patients with immunosuppression.

*Empiric antibiotic regimens. Normal renal function*

Target Pathogens: *S.aureus* and streptococci.

- One of the following oral antibiotics
- Amoxicillin-clavulanate 1 g every 8 h
- Cephalexin 500 mg every 6 h
- In patients at risk for CA-MRSA including immunocompromised status, personal or household contact with MRSA infection or colonization in the past 12 months, with prior antibiotic use for 5 days during the last 90 days or who do not respond to first-line therapy add one of the following oral antibiotics
- Minocycline 100 mg every 12 h
- Doxycycline 100 mg every 12 h
- Trimethoprim and Sulfamethoxazole 160/800 mg every 12 h

or

- In patients with beta-lactam allergy
- Clindamycin 300 mg every 8 h

In cases of recurrent skin abscess, it is necessary to look for the presence of foreign materials and identify and correct local factors that may cause recurring infection. For recurrent skin abscess bacterial culture testing should be performed to verify the causative bacteria and antibiotics susceptibility to define a targeted therapy.

If an abscess is treated with prolonged antibiotics without drainage, it can lead to formation of sterile pus surrounded by thick fibrous tissue. It makes a hard lump that sometimes mimics malignancy. The treatment is surgical drainage with excision of fibrous wall.

## Erysipelas

Erysipelas is a fiery red, tender, painful plaque with well-demarcated edges and is commonly caused by *Streptococcus spp.*, usually *S. pyogenes*. *S. aureus* rarely causes erysipelas.

Erysipelas is distinguished clinically from cellulitis by the following two features [6]:

- In erysipelas the lesions are raised above the level of the surrounding skin, and
- Erysipelas is characterized by a clear line of demarcation between involved and uninvolved tissue.

Streptococci are the primary cause. The role of *S. aureus*, and specifically MRSA, remains controversial.

*Treatment*

- Antibiotic therapy for 5 days. You may extend therapy up to 10 days if lack of symptom resolution at 5 days
- Use intravenous antibiotics if signs of systemic inflammation

*Empiric antibiotic regimens. Normal renal function*

- Target Pathogens: *S. aureus* and streptococci, CA-MRSA is unusual.

*Outpatient therapy or step down*

- One of the following oral antibiotics
- Amoxicillin-clavulanate 1 g every 8 h
- Cephalexin 500 mg every 6 h
- In patients at risk for CA-MRSA including immunocompromised status, personal or household contact with MRSA infection or colonization in the past 12 months, with prior antibiotic use for 5 days during the last 90 days or who do not respond to first-line therapy add one of the following oral antibiotics
- Trimethoprim and sulfamethoxazole 160/800–320/1600 mg every 12 h
- Minocycline 100 mg every 12 h
- Doxycycline 100 mg every 12 h

or

- In patients with beta-lactam allergy
- Clindamycin 300 mg every 8 h

or

*Inpatient therapy*

- One of following intravenous antibiotics
- Cefazolin 2 g every 8 h
- Amoxicillin-clavulanate 1.2/2.2 gr every 8 h

or

- In patients at risk for CA-MRSA including critically ill and immunocompromised status, personal or household contact with MRSA infection or colonization in the past 12 months, with prior antibiotic use for 5 days during the last 90 days or who do not respond to first-line therapy add one of following intravenous antibiotics
- Vancomycin 25–30 mg/kg loading dose then 15–20 mg/kg/dose every 12 h
- Linezolid 600 mg every 12 h

Because of their very low yield, blood cultures are not helpful in managing erysipelas, unless it is particularly severe. Culture by aspiration or punch biopsy is not recommended as principle for identifying the causative bacteria in typical erysipelas patients. However, in several cases including immunosuppressed patients or those with neutropenia, streptococci or *S. aureus* are not the causative bacteria. For these cases, lesion aspiration, or punch biopsy may be helpful to identify the causative bacteria and define a targeted therapy.

## Cellulitis

Cellulitis is an acute bacterial infection primarily of the dermal lymphatics and the subcutaneous tissue that most commonly affects the lower extremities, although it can affect other areas. It causes local signs of inflammation, such as warmth, erythema, pain, lymphangitis, and frequently systemic upset impact with fever and raised white blood cell count. Outpatient therapy should be recommended for patients with adherence to therapy, who do not have general signs of inflammation or hemodynamic instability.

Patients with a previous attack of cellulitis, especially involving the legs, can present recurrences. The infection usually occurs in the same area as the previous episode. edema, especially lymphedema, venous insufficiency, prior trauma (including surgery) to the area, and tinea pedis can increase the frequency of recurrences. Addressing these factors may decrease the frequency of recurrences.

The pathogens involved are streptococci and *S. aureus*. Cellulitis associated with abscesses is usually caused by *S. aureus*. In contrast, typical (non-purulent) cellulitis is most commonly caused by both streptococcal species and *S. aureus*. MRSA is an unusual cause of typical cellulitis.

In neutropenic and immunocompromised patients, Gram-negative bacteria should be considered.

*Treatment*

- Antibiotic therapy for 5 days. You may extend therapy up to 7-10 days if lack of symptom resolution at 5 days.
- Incision and drainage in purulent cellulitis

### Typical (non-purulent) cellulitis

*Empiric antibiotic regimens. Normal renal function*

- Target Pathogens: *S. aureus* and streptococci, CA-MRSA is unusual.

*Outpatient therapy or step-down*

- One of the following oral antibiotics
- Amoxicillin-clavulanate 1 g every 8 h
- Cephalexin 500 mg every 6 h
- In patients at risk for CA-MRSA including immunocompromised status, personal or household contact with MRSA infection or colonization in the past 12 months, with prior antibiotic use for 5 days during the last 90 days, with cellulitis associated with penetrating trauma especially from illicit drug use or who do not respond to first-line therapy add one of the following oral antibiotics
- Trimethoprim and sulfamethoxazole 160/800–320/1600 mg every 12 h
- Minocycline 100 mg every 12 h
- Doxycycline 100 mg every 12 h

or

- In patients with beta-lactam allergy
- Clindamycin 300 mg every 8 h

or

*Inpatient therapy*

- One of following intravenous antibiotics
- Cefazolin 2 g every 8 h
- Amoxicillin-clavulanate 1.2/2.2 gr every 8 h

or

- In patients at risk for CA-MRSA including critically ill and immunocompromised status, personal or household contact with MRSA infection or colonization in the past 12 months, with prior antibiotic use for 5 days during the last 90 days, with cellulitis associated with penetrating trauma especially from illicit drug use or who do not respond to first-line therapy one of the following intravenous antibiotics
- Vancomycin 25–30 mg/kg loading dose then 15–20 mg/kg/dose every 12 h
- Linezolid 600 mg every 12 h

In patients at risk for Gram-negative infections or severe forms who do not respond to first-line therapy consider.

Piperacillin/tazobactam 4,5 g every 6 h.

### Purulent cellulitis

Incision and drainage are recommended as primary management for abscesses with associated cellulitis. In these cases, antibiotics is generally suggested.

*Empiric antibiotic regimens. Normal renal function*

- Target Pathogen: *S. aureus* including CA-MRSA.

*Outpatient therapy or step-down*

- One of the following oral antibiotics
- Amoxicillin-clavulanate 1 g every 8 h
- Cephalexin 500 mg every 6 h

or

- In a region or a population with a high prevalence of CA-MRSA, where > 10% of clinical *S. aureus* isolates are MRSA isolates or in patients at high risk for CA-MRSA
- Trimethoprim and sulfamethoxazole 160/800–320/1600 mg every 12 h
- Minocycline 100 mg every 12 h
- Doxycycline 100 mg every 12 h

or

*Inpatient therapy*

- One of the following intravenous antibiotics
- Vancomycin 25–30 mg/kg loading dose then 15–20 mg/kg/dose every 12 h
- Linezolid 600 mg every 12 h
- In patients at risk for Gram-negative infections or severe forms who do not respond to first-line therapy consider
- Piperacillin/tazobactam 4,5 g every 6 h.

Culture is not recommended as principle for identifying the causative bacteria in cellulitis patients. However, in immunosuppressed patients or those with neutropenia or in severe forms associated with systemic signs of inflammation or that do not respond to first-line therapy, culture may be helpful to identify the causative bacteria and define a targeted therapy.

Cellulitis in following situations can be life-threatening thus requiring early diagnosis and prompt intervention:

*Orbital cellulitis* The infection usually spreads from paranasal sinuses and the patient presents with proptosis, chemosis, ophthalmoplegia and diminished vision due to pressure on optic nerve. Uncontrolled infection may have intra-cranial extension leading to cavernous sinus thrombosis and meningitis. Early intravenous empirical antibiotic therapy is required. Surgical drainage is required in progressive disease to prevent loss of vision.

*Ludwigs angina* It is cellulitis of submandibular region occurring deep to deep cervical fascia leading to brawny induration in this region with edema of floor of the mouth. Untreated cases may have laryngeal edema and stridor. In case of no response to antibiotics, liberal fasciotomy of deep cervical fascia in the submandibular region is required.

## Perianal and perirectal abscesses

Perianal and perirectal abscesses are typically well-circumscribed and respond to incision and drainage.

Common sites of origin of complex abscesses may be perineal or perianal, perirectal. Antibiotic therapy should be used if systemic signs of infection are present, in immunocompromised patients, if source control is incomplete, or in cases of great abscess or with significant cellulitis. Empiric broad-spectrum antibiotic therapy with coverage of Gram-positive, Gram-negative, and anaerobic bacteria should be considered.

### Diagnosis

In patients with perianal and perirectal abscesses, the diagnosis is often based on clinical assessment and examination under anaesthesia. However, in some patients several imaging techniques may be useful.

- CT
- EUS

They should be evaluated according to the specific clinical scenario and the available technology and resources.

Radiological studies are not usually needed to diagnose a complex abscess but can be useful in some special situations. The use of imaging techniques in perianal and perirectal abscesses could be helpful in all those cases with an atypical presentation (e.g., lower back pain, severe anal pain in the absence of a fissure, urinary retention), when the physical examination suggests a supra-elevator or inter-sphincteric abscess or when there is suspicion of perianal Crohn’s disease or colonic source.

MRI has high detection rates for anorectal abscesses [31], while there is debate around the sensitivity and specificity of anal endosonography (EUS). Some studies suggest that EUS is more accurate than MRI in detecting abscesses and evaluating complex fistulas, especially when suspected of arising from underlying Crohn’s disease [32]. In contrast, others report an undisputed superiority of MRI [33]. However, access to emergency MRI is often limited, and it requires a long acquisition time. In contrast, EUS requires special skills, and its use in an awake patient in the emergency setting is almost always precluded by intense anal pain. In this scenario, CT scan offers multiple advantages, such as its short acquisition time and widespread availability. The main factor limiting CT’s accuracy is the difficulty to differentiate between a fistula tract and inflammation because the tissue characteristics are very similar [34].

*Treatment*

- Incision and drainage + antibiotic therapy for 5 days in selected patients. You may extend therapy up to 7–10 days if lack of symptom resolution at 5 days.
- In perianal and perirectal abscesses identification of eventual fistula tract, and either proceed with primary fistulotomy to prevent recurrence (only in cases of low fistula not involving the sphincter muscle) or place a draining seton for future consideration. Fistulotomy can risk continence if too extensive and placement of seton should only be performed if the tract and openings are very clear, as there is risk of creating a false internal orifice and complicating the condition.

*Empiric antibiotic regimens. Normal renal function*

- Target Pathogen: Gram-positive and Gram-negative

*Outpatient therapy or step-down*

- One of the following antibiotics
- Amoxicillin/clavulanate 1 g every 8 h

or

- In patients with beta-lactam allergy
- Ciprofloxacin 500 mg every 8 h + Metronidazole 500 mg every 8 h
- In patients at risk for CA-MRSA or who do not respond to first-line therapy add one of following oral antibiotics
- Minocycline 100 mg every 12 h
- Trimethoprim and sulfamethoxazole 160/800–320/1600 mg every 12 h
- Doxycycline 100 mg every 12 h

or

*Inpatient therapy*

- One of following intravenous antibiotics
- Ceftriaxone 2 g every 24 h + Metronidazole 500 mg every 8 h
- Cefotaxime 2 g every 8 h + Metronidazole 500 mg every 8 h
- Piperacillin/tazobactam 4,5 g every 6 h

or

- In patients with beta-lactam allergy
- Ciprofloxacin 400 mg every 8 h + Metronidazole 500 mg every 8 h
- In patients at risk for CA-MRSA or who do not respond to first-line therapy add one of following intravenous antibiotics
- Vancomycin 25–30 mg/kg loading dose then 15–20 mg/kg/dose every 12 h
- Linezolid 600 mg every 12 h

Culture is not recommended as principle for identifying the causative bacteria in perianal or perirectal abscess. However, in immunosuppressed patients or those with neutropenia or in severe forms associated with systemic signs of inflammation or that do not respond to first-line therapy, it may be helpful to identify the causative bacteria and define a targeted therapy.

## Infections developing in damaged skin

Infections developing in damaged skin are a heterogeneous group including bite wounds (animal and human bites), pressure ulcers and burn wounds.

### Bite wounds (animal and human bites)

Human bite microbiology generally includes *Streptococcus* spp*, S. aureus, Peptostreptococcus* spp*, Fusobacterium* spp and *Eikinella* spp*.*

Dog bite microbiology generally includes *Pasteurella canis, Pasteurella multocida, Bacteroides* spp*, Fusobacterium* spp*, Capnocytophaga canimorsus* and *S. aureus*.

Cat bite microbiology generally includes *Pasteurella* spp*, Capnocytophaga* spp*, Bartonella henselae* (Cat-scratch diseases should be treated with azithromycin 500 mg day 1, 250 mg/day for the next 4 days*), S. aureus, Bacteroides* spp and *Fusobacterium* spp*.*

If managed incorrectly, these infections can develop into more complicated SSTIs.

*Treatment*

- Irrigation of the wound and debridement of necrotic tissue
- Antibiotic prophylaxis as principle is not recommended. It is recommended in selected patients.
- Antibiotic therapy in selected patients for 5 days. You may extend therapy up to 7–10 days if lack of symptom resolution at 5 days.
- Tetanus prophylaxis in bite wounds

Irrigation of the wound and debridement of necrotic tissue are the most important factors in preventing infection and can substantially decrease the incidence of invasive wound infection. Primary wound closure is not recommended for wounds, with the exception of those to the face, which should be managed with copious irrigation, cautious debridement, and pre-emptive antibiotics.

The comprehensive meta-analyses of Medeiros et al. in the Cochrane Database [35] demonstrated no evidential basis for reducing the infection rate by prophylactic antibiotics, except for selected cases. Despite the poor state of the evidence wounds that are moderate to severe, have associated crush injury, have associated edema (either preexisting or subsequent), that are on the hands or in proximity to a bone or a joint, or that are in compromised hosts should receive 3–5 days of antibiotic therapy [36–39].

A broad-spectrum antibiotic effective against aerobic and anaerobic organisms is required, if systemic signs of infection or severe cellulitis arise.

Human bites that have broken the skin and drawn blood can theoretically transmit hepatitis B, C, and HIV [40]. A patient who has sustained a bite should always confirm that their tetanus booster is up to date [40].

### Pressure ulcers

Pressure ulcers, also known as bedsores, decubitus ulcers and pressure injuries, are localized areas of tissue necrosis developing when soft tissue is compressed between a bony prominence and an external surface for a prolonged period of time. They are an important problem in critically ill patients, older adults, and in persons with spinal cord injury and are one of the most common types of complex wound.

Pressure ulcers can offer an ideal environment for microbial colonization. This is especially true for those pressure ulcers that may be particularly exposed to Gram-negative and Gram-positive bacterial contamination from fecal material. However, all wounds can be colonized by micro‐organisms without leading to adverse events.

Standard care for adults with pressure ulcers includes a correct prevention and management [41].

Prevention of pressure ulcer formation is directed at alleviating the risk factors for the individual patient, and is primarily focused on minimizing episodes of prolonged pressure either by placing appropriate padding at pressure points or by frequent patient repositioning.

Debridement of devitalized tissue and biofilm and abscess drainage are necessary in the treatment of pressure ulcers. In cases where there is a significant amount of necrotic tissue, performing the initial debridement in the operating room allows for a more definitive procedure. Subsequent debridements are then more easily managed at the bedside.

Dressings should be chosen depending on the wound being treated. None of the dressings has been shown to have any superiority, and the choice of dressing should depend on the type of wound being treated [42].

Any open pressure ulcer is superficially contaminated with environmental flora. However, it is important to prevent added contamination if the wound is near the fecal stream as in ischial or sacral pressure ulcers. Systemic antibiotics should be administered only when there is clinical evidence of infection.

*Treatment*

- Prevention by pressure redistribution devices such as high‐specification foam mattresses or cushions, or both and by frequent patient repositioning
- Debridement of devitalized tissue and biofilm and abscess drainage
- Appropriate selection of dressings and topical agents
- Routine use of systemic antibiotics is not currently recommended for the treatment of uninfected pressure ulcers. Systemic antibiotics should be administered only when there are systemic signs of inflammation (serious infection), spreading cellulitis (deep skin infection) or underlying osteomyelitis.
- Medical and nutritional patient optimization

### Burn wounds

Burns are one of the most common and devastating forms of trauma. Patients with serious thermal injury require immediate specialized care in order to minimize morbidity and mortality.

Although more patients with burns die of pneumonia than of burn wound infection, burn wound infection remains an important infectious complication in burn population. Treatment of burn wounds has always been a difficult medical problem and many different methods have been used to treat such injuries, locally. Early excision and grafting of the burn, is the most important operation during the patients' hospital course. Early burn debridement is vital to the overall survivability and outcome of burn patients. Eschars and blisters need to be excised and/or opened as soon as possible to cease the inflammatory cascade causing secondary damage. An important step of prevention and treatment of burn wound infections is application of dressings and topic antimicrobial agent, which act on biofilms and prevent the wound infection [43]. The selection and application of burn wound dressings and topical agents depends on the nature and extent of the burn wound. Local burn wound care aims to protect the wound surface, maintain a moist environment, promote burn wound healing, limiting burn wound progression while minimizing discomfort for the patient. Topical antimicrobial agents should be used in conjunction with appropriate basic wound care.

The various clinical challenges in treating acute thermal injuries include balancing the many factors that affect wound healing to reduce the risk of infection, the time to wound closure, and the overall time to functional recovery. The treatment of burn wounds has evolved over several decades through clinical and preclinical research. Significant advancements have been made in patient care, including tracking wound healing, developing novel graft and coverage options, controlling inflammation and optimizing dietary needs [44].

*Treatment*

- Early initiation of dressings and effective topical antimicrobial therapy
- Daily inspection of the wounds by a qualified surgeon or wound care expert
- Early excision of all full thickness and deep partial thickness burns
- Systemic antibiotic for infected wounds
- Graft and coverage options

*Empiric antibiotic regimens. Normal renal function*

- Target Pathogen: Gram-positive and Gram-negative

*Outpatient therapy or step-down*

- One of the following antibiotics
- Amoxicillin/clavulanate 1 g every 8 h

or

- In patients with beta-lactam allergy
- Ciprofloxacin 500 mg every 12 h + Metronidazole 500 mg every 8 h
- *First-generation cephalosporins, such as cephalexin, penicillinase-resistant penicillins, macrolides such as erythromycin, and clindamycin, all have poor *in vitro* activity against Pasteurella multocida and should be avoided in animal bites.*
- In patients at risk for CA-MRSA or who do not respond to first-line therapy add
- One of following oral antibiotics
- Minocycline 100 mg every 12 h
- Trimethoprim and sulfamethoxazole 160/800–320/1600 mg every 12 h
- Doxycycline 100 mg every 12 h

or

*Inpatient therapy*

- One of following intravenous antibiotics
- Ceftriaxone 2 g every 24 h + Metronidazole 500 mg every 8 h
- Cefotaxime 2 g every 8 h + Metronidazole 500 mg every 8 h
- Piperacillin/tazobactam 4,5 g every 6 h

or

- In patients with beta-lactam allergy
- Ciprofloxacin 200 mg every 8 h + Metronidazole 500 mg every 8 h
- In patients at risk for CA-MRSA or who do not respond to first-line therapy add
- Vancomycin 25–30 mg/kg loading dose then 15–20 mg/kg/dose every 12 h
- Linezolid 600 mg every 12 h

## Necrotizing infections

NSTIs are life-threatening, invasive, soft-tissue infections with a necrotizing component involving any or all layers of the soft-tissue compartment, from the superficial dermis and subcutaneous tissue to the deeper fascia and muscle.

The vicious cycle of fulminant infection, toxin production, cytokine activation, micro thrombosis and ischemia, tissue dysfunction and death, and in turn, greater dissemination of infection is central to the rapidly progressive necrosis seen in NSTIs and differentiates it from that of the other SSTIs.

NSTIs have been described according to their anatomical locations (i.e., Fournier’s gangrene) and the depth of infections: dermal and subcutaneous components (necrotizing cellulitis), fascial component (necrotizing fasciitis), and muscular components (necrotizing myositis). However, resolution of these nomenclature issues requires a consensus among international infectious disease physicians, surgeons, and intensivists, and probably, these various methods of classification are not clinically useful.

Although many specific variations of NSTIs have been described, the initial approach to diagnosis, antibiotic treatment, and surgical intervention is similar for all forms. Identifying those infections needing immediate aggressive management is more important than determining the specific variant [8].

### Diagnosis

Conditions associated with NSTIs include diabetes mellitus, renal insufficiency, arterial occlusive disease, intravenous drug abuse, body mass index (BMI) > 30 kg/m^2^, age < 65 years, liver disease, immunosuppression also in patients having tuberculosis and viral infections, recent surgery and traumatic wounds or incision of the skin, including minor lesions like insect bites and injections sites [45, 46].

Diabetic patients exhibit impaired wound healing and increased susceptibility to infection, which may affect the course of SSTIs. It is thus reasonable to speculate that this chronic, debilitating disease contributes to a more serious nature of NSTIs. Diabetes mellitus is the most common co-morbidity associated with NSTIs. Up to 44.5% of patients with this condition are diabetic [45, 46].

Patients with diabetes generally present with polymicrobial and have poorer outcomes.

Delay in diagnosis and delay in treatment of these infections increase the risk of mortality.

The initial differential diagnosis between cellulitis and NSTI that requires prompt operative intervention may be difficult. Most cases of NSTI are initially diagnosed as cellulitis. However, timely diagnosis is critical since time to operative debridement is an important determinant of outcome in NSTIs [47–50].

Patients with NSTI usually present with severe pain, which is out of proportion to the physical findings:

*Local signs*

- Edema
- Erythema
- Severe and crescendo pain out of proportion
- Skin bullae or necrosis (at a later stage)
- Swelling or tenderness
- Crepitus

The triad of swelling, erythema, and disproportionately severe pain should raise the suspicion of NSTI.

*Systemic signs*

- Fever
- Tachycardia
- Hypotension
- Shock

A rapidly progressive SSTI should be treated as an NSTI, from the beginning. The clinical picture may worsen very quickly, sometimes during a few hours. Clinical findings were reviewed by Goh et al. after a systematic literature search that identified swelling (present in 81% of NSTI cases), pain/tenderness (79%), erythema (71%), warmth (44%), bullae (26%), skin necrosis (24%), and crepitus (20%) as the most frequently encountered signs [45]. Fever was present in 40% of patients and hypotension in 21%, although the frequency of associated organ failures varies widely between studies.

Laboratory tests are not highly sensitive or specific for NSTIs. A rapidly progressive soft-tissue infection should be treated as a necrotizing infection from the beginning. The clinical picture may worsen very quickly, sometimes during a few hours.

To predict the presence of NSTI, the Laboratory Risk Indicator for Necrotizing infection (LRINEC) score was proposed (Table 1). LRINEC score assigns points for abnormalities in six independent variables: serum C-reactive protein level (> 150 mg/L), white blood cell (WBC) count (> 15,000/μL), hemoglobin level (< 13.5 g/dL), serum sodium level (< 135 mmol/L), serum creatinine level (> 1.6 mg/dL [142 mmol/l]), and serum glucose level (> 180 mg/dL [10 mmol/l]). With a score of 8 or higher, there is a 75% risk of an NSTI.

**Table 1:** Lrinec score

Variable (units)	Score points
C-reactive protein (CRP) (mg/L)	
< 150	0
≥ 150	4
White blood cell count (per mm^3^)	
< 15	0
15–25	1
> 25	2
Hemoglobin (g/dl)	
> 13.6	0
11–13.5	1
< 10.9	2
Serum sodium (mmol/L)	
≥ 135	0
< 135	2
Serum creatinine (mg/dl)	0
≤ 1.6	0
> 1.6	2
Serum glucose (mg/dl)	
≤ 180	0
> 180	1

Subsequent evaluation of the LRINEC score has demonstrated conflicting results. Several studies have assessed the utility of LRINEC for the early diagnosis of necrotizing infections [51–54].

Recent evidence has demonstrated that it lacks the sensitivity to be a useful adjunct for diagnosing NSTIs [55]. The LRINEC score has poor diagnostic accuracy for NSTI, and a low score does not rule out the diagnosis.

### Imaging

The diagnosis of NSTIs is primarily clinical. However, radiologic imaging may be able to provide useful information when the diagnosis is uncertain. A plain X-ray should not be used to rule out NSTI. However, it is important that if clinical suspicion of NSTI is high, radiologic imaging must neither delay nor deter surgery, because in this setting an early surgical debridement is essential to decrease mortality.

- CT
- MRI
- US

CT has a higher sensitivity than plain radiography in identifying early NSTIs. Findings consistent with necrotizing infections are fat stranding, fluid and gas collections that dissect along fascial planes, and gas in the involved soft tissues. Additionally, fascial thickening and non-enhancing fascia on contrast CT suggests fascial necrosis [56–58]

MRI has been considered the imaging modality of choice for necrotizing fasciitis [53]. However, MRI may be difficult to perform under emergency conditions and is not recommended as the first-choice imaging technique.

US has the advantage of being rapidly performed at the bedside and may help differentiate simple cellulitis from NSTIs [45], but again is not considered highly accurate nor definitive in most settings. Point-of-care US (POCUS) can improve diagnose accuracy for NSTI when used in combination with clinical evaluation as it is increasingly available, fast and can be performed at the bedside [59]. The main ultrasound findings are summarized in loss of the normal tissue architecture to a “cobblestone” appearance, with irregularity and thickening of the fascia, abnormal fluid collections along the fascia, seen as hypoechogenic zones, and, in more advanced cases, the presence subcutaneous air, defined by hyperechogenic foci with a posterior dirty acoustic shadowing.

Moreover, POCUS can show evidence of gas in the soft tissue, indicative of advanced disease and a marker of worse prognosis. The presence of a thickened fascia can make it difficult to differentiate the underlying structures. Yet, there is always the possibility of comparing with another similar unaffected structure, usually the other limb. Ultrasound can also be helpful to guide fluid drainage if a collection is present and rule out deep vein thrombosis.

A systematic review of the literature and meta-analysis including 23 studies was carried out to establish and compare the accuracy of physical examination, imaging, and LRINEC score in diagnosis of NSTIs [60]. Twenty-three studies were included in the analysis with a total of 5982 patients. Of physical examination signs, pooled sensitivity and specificity for fever was 46.0% and 77.0% respectively, for hemorrhagic bullae 25.2% and 95.8%, and for hypotension 21.0% and 97.7%. CT had sensitivity of 88.5% and specificity of 93.3%, while plain radiography had sensitivity of 48.9% and specificity of 94.0%. Finally, LRINEC ≥ 6 had sensitivity of 68.2% and specificity of 84.8%, while LRINEC ≥ 8 had sensitivity of 40.8% and specificity of 94.9%.

*Invasive diagnosis*

- Fascial biopsy with frozen section
- Finger test

Fascial biopsy with frozen section has been suggested to achieve earlier diagnosis of NSTIs [61]. However, a frozen-section biopsy is not very practical and requires the availability and experience of pathologists, and the time taken to carry out and analyze the sample could be used for debridement [62]. The Finger test is another adjunct method described for diagnosing NSTIs. It is performed under local anesthesia. A 2-cm incision is made down to the deep fascia. Minimal tissue resistance to finger dissection (positive Finger test), the absence of bleeding, the presence of necrotic tissue, and/or murky and grayish (“dishwater”) fluid following incision all suggests the diagnosis of NSTI [63].

*Treatment*

- Surgical source control as soon as possible within 6 h after admission. Delay in early surgical increases mortality.
- Appropriate and effective debridement techniques. Skin-sparing debridement techniques focusing on tissue directly involved in necrosis.
- Re-explorations should be repeated until the time when very little or no debridement is required.
- Empiric antibiotic therapy optimizing Pharmacokinetics (PK) and Pharmacodynamics (PD) targets.
- Deep samples collected at the interface between healthy and necrotized tissues during initial debridement and blood cultures allow the identification of causative pathogens in most cases.
- De-escalation of antibiotic therapy be based on clinical improvement, cultured pathogens, and results of rapid diagnostic tests where available
- (Organ) supportive measures
- Hyperbaric oxygen therapy where it is available
- Intravenous immunoglobulin (IVIG) in patients with streptococcal NSTIs

Early source control, antibiotic therapy, and (organ) supportive measures are the cornerstone of treatment in patients with sepsis or septic shock caused by NSTIs.

Early surgical debridement with complete removal of necrotic tissue, including potential major amputation is essential to decrease mortality and other complications in patients with NSTIs.

While antibiotic therapy, resuscitation and critical care evaluation are necessary in the treatment of patients presenting with NSTIs, the mainstay of therapy remains surgical treatment. Once the diagnosis of NSTI is suspected, early consultation with a surgeon is always warranted. Delay in the identification or early surgical management of these infections clearly increases mortality [8]. Debridements should be always performed in operating room where the best exposure and examination of the wound in a pain-free environment can be performed. Typical intraoperative findings in progressive NSTI are coagulative necrosis of the tissues and the subcutaneous layer with muddy, dishwater-like fluid.

Deep samples collected at the interface between healthy and necrotized tissues during initial debridement and blood cultures are crucial, allowing for the identification of causative pathogens in most cases.

In order to review the literature concerning the timing of surgery in relation to mortality and amputation in patients with NSTIs a systematic search and meta-analysis was recently published. A total of 109 studies, with combined 6051 NSTI patients, were included [27]. Of these 6051 NSTI patients, 1277 patients died (21.1%). A total of 33 studies, with combined 2123 NSTI patients, were included for quantitative analysis. Mortality was significantly lower for patients with surgery within 6 h after presentation compared to when treatment was delayed more than 6 h. Surgical treatment within 6 h resulted in a 19% mortality rate compared to 32% when surgical treatment was delayed over 6 h. Average mortality rates reported remained constant (around 20%) over the past 20 years. Early surgical debridement lowers the mortality rate for NSTI with almost 50%. Thus, a sense of urgency is essential in the treatment of patients with NSTIs.

Early surgical debridement with complete removal of necrotic tissue is essential to decrease mortality and other complications in patients with NSTIs [64]. It is the most important determinant of outcome in patients with NSTIs and should be performed as soon as possible, but at least within the first 6 h after admission.

Skin grafting and extensive rehabilitation are necessary to mitigate disfigurement after invasive debridements, limited joint mobility, and chronic pain. When debridement focuses only on tissue directly involved in necrosis, viable skin and subcutaneous tissue can remain in place despite wide debridement of deeper tissue planes [28, 29].

Skin-sparing debridement techniques have been described in the literature. Relative to traditional debridement, skin-sparing debridement for source control of NSTI results in significantly more wounds closed completely by delayed primary suture of existing skin flaps and a significantly lower overall wound percentage closed by skin graft, while demonstrating equivalent efficacy of source control and a similar low mortality rate.

Scheduled re-explorations should be done at least every 12–24 h after the initial operation or sooner if clinical local or systemic signs of worsening infection become evident, as well as with worsening laboratory parameters (WBC count, C Reactive Protein and Procalcitonin). Re-explorations should be repeated until the time when very little or no debridement is required.

After debridement and once the wound is stable, the subsequent use of negative pressure therapy allows reduction of the wound surface, extraction of wound exudate and cell residues, as well as induction of granulation.

Microbiologically, NSTIs have been classified as either type 1 (polymicrobial) or type 2 (mono-microbial) or type 3 (gas gangrene). Occasionally in immunocompromised patients, NSTIs may be also caused by mycotic species.

Type I NSTIs is a polymicrobial infection involving aerobic and anaerobic organisms. It is associated with surgical procedures involving the bowel or penetrating abdominal trauma, with infections developed in damaged skin, such as decubitus ulcer or animal bites, with infections at the site of injection in injection drug users, or with a perianal, prostate or vulvovaginal abscess [6].

Type I infection may be often associated with gas in the tissue and thus is difficult to distinguish from gas gangrene.

Type II NSTI is a mono-microbial infection. In the monomicrobial form, the most common pathogens are anaerobic streptococci and *S. aureus*. Staphylococci and streptococci can occur simultaneously. Most infections are community-acquired and present in the limbs, with approximately two-thirds of cases in the lower extremities. *Vibrio vulnificus* and *Aeromonas hydrophila* are the most common Gram-negative bacteria causing type II NSTIs.

Gas gangrene (clostridial myonecrosis), or type III NSTI, is an acute infection by clostridium or bacillus of healthy living tissue that occurs spontaneously or as a result of traumatic injury.

Occasionally in immunocompromised patients, NSTIs may also be caused by mycotic species. Empiric coverage against fungi should be started in high-risk patients.

Since it is impossible to exclude with certainty a polymicrobial NSTI, an aggressive broad-spectrum empiric antimicrobial therapy should initially be selected to cover Gram-positive, Gram-negative, and anaerobic organisms until culture-specific results and sensitivities are available. An acceptable empiric antibiotic regimen should always include antibiotics, which cover MRSA with the additional benefit of inhibiting invasive hamolytic streptococci virulence proteins.

Selection of antibiotics that inhibit toxin production may be helpful, particularly in those patients who have evidence of toxic shock syndrome (TSS), potentially present in patients who have streptococcal and staphylococcal infections [65–67]. Protein cytotoxins, such as superantigens, play an important role in the pathogenesis of various staphylococcal and streptococcal infections, and toxin production should be considered when selecting an antimicrobial agent for Gram-positive pathogens. Linezolid and clindamycin play an important role because they may significantly inhibit exotoxin production from Gram-positive pathogens [8]. Culture-specific results and sensitivities can direct both broadening of antibiotic regimen if it is too narrow and a de-escalation if it is too broad particularly in critically ill patients where de-escalation strategy is one of the cornerstones of antimicrobial stewardship programs [8].

In the absence of definitive clinical trials, antibiotic therapy should be administered until further debridement is no longer necessary.

In patients with NSTIs the antibiotic dosing regimen should be established depending on host factors and properties of antibiotic agents [68]. The achievement of appropriate target site concentrations of antibiotics is essential to eradicate the pathogens. Suboptimal target site concentrations may have important clinical implications, and may explain therapeutic failures, in particular, for bacteria for which in vitro MICs are high. One major consequence of septic shock, affecting half of patients with NSTIs, is the intense vasodilation and extravasation of fluid into the interstitial space from endothelial damage and capillary leakage. This phenomenon is commonly described as ‘third spacing’. Moreover, to contrast hypotension, large volumes of resuscitation fluids are administered distributing into interstitial space, thereby significantly increasing interstitial volume [69].

The “dilution effect”, also called the ‘third spacing’ phenomenon, must be considered when administering hydrophilic agents such as β-lactams, aminoglycosides, and glycopeptides, which selectively distribute to the extracellular space [69]. Low plasma antibiotic levels can contribute to lower-than-expected antibiotic concentrations in soft tissue with potentially reducing antibiotic delivery to the target tissues. In addition, hypoalbuminaemia is a common condition in patients with NSTIs. With low albumin concentrations, an increase in the unbound fraction of antibiotics can occur. The unbound fraction of antibiotics is not only available for elimination, but also for distribution to the target tissue.

Knowledge of the pharmacokinetic and pharmacodynamic antibiotic properties may provide a more rational determination of optimal dosing regimens in terms of the dosing interval. Optimal use of the pharmacokinetic/pharmacodynamic relationship of antibiotics is important to obtain an optimal target site concentration. It is related to the concept of time-dependent versus concentration-dependent killing [69]. Beta-lactams exhibit time-dependent activity and exert optimal bactericidal activity when drug concentrations are maintained above the MIC. Therefore, it is important that the serum concentration of beta-lactam agents exceeds the MIC for appropriate duration of the dosing interval. Higher frequency dosing, prolonged infusions and continuous infusions have been utilized to achieve this effect.

Finally, tissue penetration is also an important aspect because high concentrations at the site of infection can optimize the effect of antibiotics. One recent study included 11 obese patients with severe SSTI, of whom 9 had NSTI, and evaluated the pharmacokinetics of linezolid. Although linezolid has an excellent soft tissue distribution, the probability of target attainment for this antibiotic was low using the standard dosing of 600 mg every 12 h [70].

Despite significant advancements in critical care management and improved knowledge regarding NSTIs, mortality remains elusively high. Adjunctive and less conventional treatment options have been explored to improve outcomes in this group of patients. Hyperbaric oxygen (HBO) is one of these modalities [71]. The role of HBO as an adjunctive treatment has been debated, and no prospective randomized clinical trials have been published nor valid research evidence produced regarding the effects of HBO therapy on wound healing.

HBO could be considered, if available, but it should not interfere with nor delay the standard treatment, including aggressive ICU support if required. Furthermore, the patient should not be transferred to carry out HBO therapy, thereby delaying critical care.

Intravenous immunoglobulin therapy has been postulated to improve outcomes in a selected population of patients with NSTIs. Most reported studies evaluated its use for invasive GAS infections, including GAS-related NSTIs with streptococcal toxic shock syndrome (STSS) [72] with variable results.

Finally, intensive care for hemodynamic and metabolic support should be performed as soon as possible. Disease severity, reflected by the severity of illness scores such as the APACHE II [73] or hypotension and/or vasopressor need [74], are risk factors for mortality.

Patients with NSTIs are very complex and may lose fluids, proteins, and electrolytes through a large surgical wound. In addition, hypotension is caused by vasodilation induced by the systemic inflammatory response syndrome to infection and cytotoxins [75]. Fluid resuscitation and analgesia are the mainstays of support for patients with advanced sepsis, usually combined with vasoactive amines associated with mechanical ventilation and other organ function support, if needed. No ideal fluid has been proven: however, resuscitation therapy must be prompt and immediate as in any type of shock.

Recently the new guidelines of Surviving Sepsis Campaign have been published [76]. Below a brief summary of what is pertinent to NSTIs is reported.

- As soon as possible after diagnosing sepsis (organ dysfunctions) associated with a NSTI, administer a 1-L bolus of a balanced crystalloid solution over 30 min. In hypotensive patients or those with an elevated serum lactate level additional fluid should be administered to achieve 30 ml/kg of initial volume resuscitation. This should be administered within 3 h.
- In patients who do not achieve a MAP ≥ 65 mmHg with initial volume resuscitation within one hour, start a norepinephrine infusion and titrate as needed. This can initially be administered through a peripheral IV while central venous access is being obtained.
- Simultaneously, administer within 1-h broad spectrum antimicrobial agent(s) to cover potential pathogens.
- If the norepinephrine infusion increases to ≥ 15 mg/min, add low dose vasopressin at infusion rate of 0.03 U/min. Do not increase this dose of vasopressin.
- Start low dose steroids (hydrocortisone 50 mg q 6 h) in patients requiring ongoing high doses of norepinephrine and vasopressin to achieve MAP ≥ 65 mmHg.
- Additional fluid resuscitation (beyond the initial 30 ml/kg) will likely be needed but should be based on the assessment that the patient will be fluid responsive.
- Patients with impaired cardiac function should inotropic agent started. Dobutamine is the preferred agent but will cause hypotension in hypovolemic patients.

### New agents to treat NSTIs

Reltecimod (previously known as AB103 or p2TA), a peptide derived from the T-cell receptor CD28, modulates the host immune response by targeting the co-stimulatory pathway, which is essential for the induction of multiple pro-inflammatory cytokines. Consequently, reltecimod has demonstrated beneficial effects against different bacterial infections such as NSTIs.

A randomized, double-blind, placebo-controlled trial of single dose reltecimod (0.5 mg/kg) administered within 6 h of NSTI diagnosis was recently published [77]. Reltecimod was associated with improved resolution of organ dysfunction and hospital discharge status. Further studies are warranted to establish the real efficacy in clinical practice.

### Wound management after source control

The rapidly spreading infection followed by aggressive surgical intervention and repeated debridements creates challenges for wound management.

Negative pressure wound therapy (NPWT) refers to wound dressing systems that continuously or intermittently apply sub-atmospheric pressure to the surface of a wound. NPWT has become a popular treatment modality for the management of many acute and chronic wounds [8]. In the setting of necrotizing infections once the necrosis is removed, NPWT can help wound healing physiologically. The negative pressure leads to an increased blood supply, increasing tissue perfusion, reducing edema, absorbing fluids and exudates, inhibiting infection, and finally drying the wound and thus the migration of inflammatory cells into the wound. Additionally, it promotes and accelerates the formation of granulation tissue by the removal of bacterial contamination and exudates. A modification of the original system added intermittent automated instillation of topical wound irrigation solutions to traditional NPWT. It, named NPWTi, has been shown to be effective in the treatment of a variety of complex wounds. NPWTi has been shown to reduce biofilms present in wounds helping heal clinically infected wounds.

As alternative the plastic surgical closure and reconstruction an optimal option. However, it needs a strict collaboration with the plastic surgeons. Although skin grafting may fulfill this role, techniques higher on the reconstructive ladder, including local, regional and free flaps, are sometimes undertaken [78].

*Empiric antibiotic regimens. Normal renal function*

The initial empirical antibiotic regimen should comprise broad-spectrum drugs, including anti-MRSA and anti-Gram-negative coverage. Antitoxin active antibiotics such as clindamycin or linezolid should be included in the empirical antibiotic regimen to treat NSTIs.

*In stable patients*

- One of the following antibiotics
- Amoxicillin/clavulanate 1.2/2.2 g every 8 h
- Ceftriaxone 2 g every 24 h + Metronidazole 500 mg every 8 h
- Cefotaxime 2 g every 8 h + Metronidazole 500 mg every 8 h
-  + 
- Clindamycin 600–900 mg every 8 h

*In unstable patients*

- One of the following antibiotics
- Piperacillin/tazobactam 4.5 g every 6 h
- Meropenem 1 g every 8 h
- Imipenem/Cilastatin 500 mg every 6 h
-  + 
- One of the following antibiotics
- Linezolid 600 mg every 12 h
- Tedizolid 200 mg every 24 h

or

- Another anti-MRSA-antibiotic as
- Vancomycin 25–30 mg/kg loading dose then 15–20 mg/kg/dose every 8 h
- Daptomycin 6–8 mg/kg every 24 h *
- Telavancin 10 mg/kg every 24 h
-  + 
- Clindamycin 600–900 mg every 8 h

*Approved at the dosage of 4–6 mg/kg/24 h, it is currently used at higher dosages.

## Fournier’s gangrene

Fournier’s gangrene (FG) is a severe type of NSTI involving the genital area and or perineum.

The origin of the infection is identifiable in the majority of cases and is predominantly from anorectal, genito-urinary or local cutaneous sources. The aggressive nature of the infection requires early recognition and immediate surgical intervention.

Patients with FG usually present with severe pain, which is out of proportion to the physical findings.

*Diagnosis*

Local signs:

- edema.
- Erythema.
- Severe and crescendo pain out of proportion.
- Skin bullae or necrosis (at a later stage).
- Swelling or tenderness.
- Crepitus.

Systemic signs:

- Fever.
- Tachycardia.
- Hypotension.
- Shock.

Fournier’s Gangrene severity index (FGSI) is a standard score for predicting outcome in patients with FG and is obtained from a combination of physiological parameters at admission, including temperature, heart rate, respiration rate, sodium, potassium, creatinine, leukocytes, haematocrit, and bicarbonate. An FGSI score above 9 has been demonstrated to be sensitive and specific as a mortality predictor in patients with Fournier’s gangrene [79–82] (Table 2).

**Table 2:** Fournier’s Gangrene severity index

Physiological variables	+ 4	+ 3	+ 2	+ 1	0	+ 1	+ 2	+ 3	+ 4
Temperature (C)	> 41	39–40	–	38–39	36–38.4	34–35.9	32–33.9	30–31.9	< 29.9
Heart rate (bpm)	> 180	140–179	110–139	–	70–109	–	55–69	40–54	< 39
Respiratory rate	> 50	35–49	–	25–34	12–24	10–11	6–9	–	< 5
Serum K + (mmol/L)	> 7	6–6.9	–	5.5–5.9	3.5–5.4	3–3.4	2.5–2.9	–	< 2.5
Serum Na^+^ (mmol/L)	> 180	160–179	155–159	150–154	130–149	–	120–129	110–119	< 110
Serum creatinine (mg/1000 ml) (× 2 for acute renal failure)	> 3.5	2–3.4	1.5–1.9	–	0.6–1.4	–	< 0.6	–	–
Hemotocrit (%)	> 60	–	50–59	46–49	30–35	–	20–29	− < 20	
WBC (mm^3^)	> 40	–	20–39.9	15–19	3–14.9	–	1.2.9	–	< 1
Serum bicarbonate venous (mmol/L)	> 52	41–51	–	32–40	22–31	–	18–21	15–17	< 15

*Treatment*

- Surgical source control as soon as possible. Re-explorations should be repeated until the time when very little or no debridement is required.
- Diverting colostomy or rectal diversion devices
- Antibiotic therapy
- (Organ) supportive measures

Surgical debridement must be early and aggressive to halt the progression of infection. Cultures of infected fluid and tissues should be obtained during the initial surgical debridement, and the results used to tailor specific antibiotic management. Radical surgical debridement of the entire affected area should be performed, continuing the debridement into the healthy-looking tissue [83, 84].

In the setting of FG, diverting colostomy has been demonstrated to improve outcomes but are poorly controlled studies that do not consider the morbidity and impact of the colostomy and its potential reversal.

A transverse loop colostomy is preferred because it yields solid and formed stools with little contamination of the surrounding skin. The abdomen above the umbilicus is ideal because FG often extends into the lower abdominal wall [85].

It may help in minimizing bacterial load in the perineal wound, thus controlling infection [86]. Diverting colostomy does not eliminate the necessity of multiple debridements nor reduces the number of these procedures [86]. Diverting colostomy should be avoided as much as possible, mainly when there are other methods to avoid wound contamination. Recently, rectal diversion devices have been marketed. They are silicone tubes designed to divert fecal matter in patients with diarrhoea, local burns, or skin ulcers. The devices protect the wounds from fecal contamination and reduce, in the same way a colostomy does, the risk of skin breakdown and repeated inoculation with colonic microbial flora. fecal diversion tubes can be used in combination with negative pressure wound therapy to effectively isolate the wound from fecal contamination [87]. Most FG infections are polymicrobial, and since it is impossible to exclude with certainty a polymicrobial necrotizing infection, an aggressive broad-spectrum empiric antimicrobial therapy should initially be selected to cover Gram-positive, Gram-negative, and anaerobic organisms until culture-specific results and sensitivities are available. An acceptable empiric antibiotic regimen should always include antibiotics, which cover MRSA with the additional benefit of inhibiting invasive GAS virulence proteins. For the treatment of MRSA, we refer to the previous paragraphs.

To treat Gram-negative bacteria, the use of piperacillin-tazobactam in the setting without the high local prevalence of ESBL-producing Enterobacteriaceae optimizing pharmacokinetic / pharmacodynamic parameters is appropriate. Carbapenems, administered in adequate dosage, including meropenem, imipenem-cilastatin, or doripenem, may be used in the settings with a high local prevalence of ESBL-producing Enterobacteriaceae. Culture-specific results and sensitivities can direct both broadenings of antibiotic regimen if it is too narrow and the de-escalation if it is too broad, particularly in critically ill patients where de-escalation strategy is one of the cornerstones of antimicrobial stewardship programs.

*Empiric antibiotic regimens. Normal renal function*

The initial empirical antibiotic regimen should comprise broad-spectrum drugs, including anti-MRSA and anti-Gram-negative coverage. Antitoxin active antibiotics such as clindamycin or linezolid should be included in the empirical antibiotic regimen to treat NSTIs.

*In stable patients*

- One of the following antibiotics
- Amoxicillin/clavulanate 1.2/2.2 g every 8 h
- Ceftriaxone 2 g every 24 h + Metronidazole 500 mg every 8 h
- Cefotaxime 2 g every 8 h + Metronidazole 500 mg every 8 h
-  + 
- Clindamycin 600–900 mg every 8 h

*In unstable patients*

- One of the following antibiotics
- Piperacillin/tazobactam 4.5 g every 6 h
- Meropenem 1 g every 8 h
- Imipenem/Cilastatin 500 mg every 6 h
-  + 
- One of the following antibiotics
- Linezolid 600 mg every 12 h
- Tedizolid 200 mg every 24 h

or

- Another anti-MRSA-antibiotic as
- Vancomycin 25–30 mg/kg loading dose then 15–20 mg/kg/dose every 8 h
- Teicoplanin LD 12 mg/kg 12-hourly for 3 doses, then 6 mg/kg every 12 h
- Daptomycin 6–8 mg/kg every 24 h *
- Telavancin 10 mg/kg every 24 h
-  + 
- Clindamycin 600–900 mg every 8 h

*Approved at the dosage of 4 mg/kg/24 h, it is currently used at higher dosages.

## Water and soil-borne necrotizing infections

*Aeromonas hydrophila* is a rod-shaped, motile, and Gram-negative bacterium. It is found in wastewater, sewage, and food. Wound infection of *A. hydrophila* in humans is the second most frequent after oral infection and is associated with traumatic events and wounds exposed to water and soil [88]. Most cases of *A. hydrophila* wound infection occur in healthy people. In particular, *A. hydrophila* wound infection is reported following natural disasters, such as the tsunami and hurricane. The wound infections due to *A. hydrophila* can progress rapidly to NSTIs [89].

*Vibrio vulnificus* is a halophilic, Gram-negative bacterium belonging to the *Vibrio genus* and Vibrionaceae family.

*V. vulnificus* can be transmitted through the consumption of contaminated fish and shellfish, and skin exposure to contaminated seawater [90]. The sensitivity of the host is crucial for the development of *V. vulnificus* infection, which commonly occurs in patients with chronic liver diseases, such as diabetes mellitus, end-stage renal disease, rheumatoid arthritis, and hemochromatosis [91]. The wound infections due to *V. vulnificus* can progress to NSTIs.

Mortality for *V. vulnificus* serious wound infections have been shown to increase with greater delays between onset of illness and initiation of antibiotic treatment and aggressive debridement. Thus, patients with a presumptive diagnosis of *V. vulnificus* should be treated immediately by antibiotics and managed aggressively by a prompt debridement and resuscitation in an intensive care unit to minimize the possible consequences of hypotension, septic shock, and the risk of multiorgan system failure.

The contact history of patients with a rapid onset of cellulitis can alert clinicians to a differential diagnosis of soft-tissue infection with *V vulnificus* (contact with seawater) or *A. hydrophila* (contact with fresh or brackish water, soil, or wood) [92].

## Gas gangrene

Gas gangrene also named clostridial myonecrosis and is another highly lethal NSTIs, caused by *Clostridium species*, with *Clostridium perfringens* being the most common [93].

Clostridial infections usually arise in traumatized tissues. However, it can also arise spontaneously. The infection involves deeper tissue such as a muscle which can lead to a rapidly spreading infection along tissue planes, and patients often present with sepsis.

*C. perfringens* causes 80–90%, of gas gangrene cases, but other species can cause infection, including *C. novyi, C. septicum, C. histolyticum, C. bifermentans, C. fallax,* and *C. sordellii* [93].

The fulminant clinical and histological features of an infection with clostridia are mediated by potent bacterial exotoxins, making clostridial myonecrosis the most rapidly spreading and lethal infection in humans.

*Clostridium spp*. can produce alpha and theta toxins that cause extensive tissue damage.

The primary toxin to mediate the effect of *C. perfringens* is alpha-toxin, a zinc metallophospholipase with phospholipase C and sphingomyelinase activity. Alpha-toxin is thought to be the major factor for tissue pathology leading to muscle necrosis and hemolysis [94]. The second major toxin is theta-toxin, a pore-forming toxin [95].

The infection can spread quickly, and within a matter of several hours, the patient may develop overwhelming shock, sepsis, and death. The infection can develop slowly over weeks or rapidly over hours depending on the oxygen tension of the tissue and the amount of organism inoculated.

Increasingly severe pain beginning within 24 h at the injury site is the first reliable clinical symptom. The skin may initially appear pale, but quickly changes to bronze, then purplish-red. The infected region becomes tense and tender, and bullae filled with reddish-blue fluid appear. Gas in the tissue, detected as crepitus or by imaging, is usually present by this late stage. Signs of systemic toxicity, including tachycardia, fever, and diaphoresis, develop rapidly, followed by shock and multiple organ failure.

Because the infection is rapidly progressive, it is important to treat patients aggressively, by early surgical debridement, antibiotics and intravenous fluid resuscitation.

## Mesh infection

Hernia repair is one of the most common surgical procedures performed globally. Mesh infection, although infrequent, is a devastating complication of mesh hernioplasties. Currently, several types of prosthetic mesh are widely used for repairing abdominal wall defects; however, there is no single universal ideal mesh. Synthetic meshes are easy to handle and well-tolerated; however, they can be potentially associated with infection when bacteria adhere to the synthetic material leading to chronic infection. Mesh infection is a challenging complication of abdominal wall defect repairs.

Surgeons should recognize the potential for any mesh to become infected and understand the risks and management strategies for mesh infection.

Mesh infections should be distinguished from superficial incisional SSIs. They occur in the early postoperative period and are not influenced by mesh implantation but can cause the infection of the mesh. Patients with deep mesh infections may present with signs of local inflammation. However, more frequently, deep mesh infections tend to be indolent and present chronic signs and symptoms. They may be initially underestimated.

The usual causative micro-organisms associated mesh infection are *S. aureus* including MRSA, *S. epidermidis* and streptococci and Gram-negative bacteria including Enterobacteriaceae. The aim of the infection prevention and control strategies including surgical antibiotic prophylaxis is to minimize bacterial count in the wound and decrease adherence to the mesh preventing biofilm production; thereby blocking the key step for mesh infection.

Bacterial adherence and biofilm formation on the surface of synthetic materials are essential steps in the sequence leading to mesh infections. The first stage of mesh infection is bacterial adherence to the prosthesis. Bacterial adhesion is the result of the interaction between the bacteria and the mesh. The result of this interaction is the formation of the bacterial biofilm [96–98]. Embedded in self-secreted extracellular polymeric substances, biofilm can provide bacteria an effective barrier against host immune cells and antibiotics. The nature of biofilm structure makes bacteria difficult to eradicate and confer an inherent resistance to antibiotics.

The management of mesh infections is challenging and always requires an individualized approach combining medical and surgical approaches. Although, several studies have demonstrated that in certain instances, non-operative strategies with conservative (non-surgical) management have been successful for salvaging a mesh [99] in many cases complete surgical removal of the mesh is suggested to reduce the risk of infection recurrence or severe complications, such as visceral adhesions and fistulae.

After removing the infected mesh, the intra-operative options are (a) no implant of a new mesh, (b) re-implantation of a new synthetic light-weight, macroporous mesh, and (c) replacement of the infected synthetic by a biological mesh [100, 101].

In 2016, Atema et al. [102] published a systematic review and meta-analysis of the repair of potentially contaminated and contaminated abdominal wall defects. In potentially contaminated hernias (CDC wound class 2), no benefit of biologic over synthetic mesh was found with comparable surgical site complication rates and a hernia recurrence rate of 9% for biologic and 9% for synthetic repair. In contaminated hernias (CDC wound class 3 and 4), most reports were on biologic mesh repair, showing high rates of surgical site complications and a hernia recurrence rate of 30%. Recurrence rates in contaminated hernias depended on whether primary fascial closure was achieved, or the repair with biologic mesh was bridging. Biologic mesh sublay repair with primary fascial closure showed lower recurrence rates than bridging repairs.

## Conclusions

SSTIs encompass a variety of pathological conditions ranging from simple superficial infections to severe necrotizing infections. The multifaceted nature of these infections has led to a collaboration among general and emergency surgeons, intensivists, and infectious diseases specialists, who have shared these evidence-based pathways.

## Data Availability

Not applicable.

## Abbreviations

ABSSSI: Acute bacterial skin and skin structure infection
CA-MRSA: Community associated methicillin-resistant *S. aureus*
HA-MRSA: Hospital associated methicillin-resistant *S. aureus*
LRINEC: Laboratory risk indicator for necrotizing infection
MRSA: Methicillin-resistant *S. aureus*
NSTIs: Necrotizing soft-tissue infections
SSIs: Surgical site infections
SSTIs: Skin and soft-tissue infections
